# A Comparative Evaluation of Shade Selection Using Digital Photography and the VITA 3D-Master Shade Guide: An In Vitro Study

**DOI:** 10.7759/cureus.105908

**Published:** 2026-03-26

**Authors:** Arvind K Singh, Shruti S Grover, Gaurav Chandra, Joy K Baishya, Shalini Pandey

**Affiliations:** 1 Department of Prosthodontics, Chandra Dental College and Hospital, Barabanki, IND

**Keywords:** digital photography, in vitro study, prosthodontics, tooth shade selection, vita 3d-master shade guide

## Abstract

Aim and objectives

Accurate shade selection is critical for achieving optimal esthetic outcomes in restorative dentistry. Digital photography has emerged as an alternative to conventional visual shade matching; however, the accuracy of smartphone (SP) cameras compared with digital single-lens reflex (DSLR) cameras remains uncertain. This study aimed to compare Commission Internationale de l'Éclairage L*a*b* (CIELAB) color coordinates obtained from SP and DSLR images with manufacturer-provided values of the VITA 3D-Master shade guide (VITA Zahnfabrik, Bad Säckingen, Germany) and to evaluate the reliability of smartphone photography for shade selection.

Methods

An in vitro study was conducted using a commercial VITA 3D-Master shade guide. Images of 26 shade tabs were captured using an iPhone 13 smartphone (Apple Inc., Cupertino, CA, USA) and a Canon EOS 700D DSLR (Canon Inc., Tokyo, Japan). Two images per shade tab were obtained under standardized daylight conditions (4000-5000 K) at a fixed distance of 18 cm against a neutral gray background, yielding a total of 104 images. Image selection was standardized based on predefined criteria of focus and exposure consistency. CIELAB color values (L*, a*, b*) were extracted using digital image analysis software. Color differences (ΔE) between photographic values and manufacturer reference values were calculated using the CIEDE2000 formula. Statistical analysis was performed using the Wilcoxon signed-rank test, with significance set at P < 0.05.

Results

Statistically significant differences were observed between SP and DSLR images in deviations of L*, a*, and b* values (P < 0.05). DSLR images demonstrated greater color accuracy, achieving 75% agreement with manufacturer reference values, whereas SP images showed 55% agreement. Mean ΔE values were lower for DSLR images, indicating improved color fidelity.

Conclusions

Within the limitations of this in vitro study, DSLR photography demonstrated greater accuracy in shade selection compared to smartphone photography. Although smartphones may serve as an accessible adjunct, they currently exhibit lower color accuracy than DSLR systems for shade determination.

## Introduction

Accurate shade selection is essential for achieving optimal esthetic outcomes in restorative dentistry. Tooth color results from the interaction of light with dental tissues and is perceived through the human visual system, making color determination a complex and inherently subjective process [1].

Traditionally, shade matching has been performed using visual comparison with standardized shade guides. Although this approach is simple and cost-effective, it is highly subjective and influenced by factors such as lighting conditions, operator experience, visual fatigue, and the polychromatic nature of teeth. Furthermore, conventional shade guides may not fully represent the entire spectrum of natural tooth color [2-4].

To enhance the accuracy and reproducibility of shade selection, objective instrumental methods such as spectrophotometers, colorimeters, and digital image analysis have been introduced. Color measurements are commonly expressed using the Commission Internationale de l'Éclairage L*a*b* (CIELAB) color system, in which L* represents lightness, a* the red-green axis, and b* the yellow-blue axis. Color differences between two samples are quantified using ΔE values, which indicate the magnitude of color discrepancy [5].

In recent years, digital photography has gained importance as a tool for shade selection and communication between clinicians and dental laboratories. Advances in imaging technology have enabled smartphones equipped with high-resolution cameras to be increasingly utilized in clinical practice due to their portability, accessibility, and ease of use. However, despite these advantages, evidence regarding the accuracy and reliability of smartphone-based shade assessment compared with digital single-lens reflex (DSLR) cameras remains limited.

Therefore, the aim of this in vitro study was to compare the CIELAB color coordinates (L*, a*, b*) obtained from smartphone and DSLR images with those of the VITA 3D-Master shade guide (VITA Zahnfabrik, Bad Säckingen, Germany) to evaluate their reliability for dental shade selection.

The primary outcome measure of this study was the mean color difference ΔE calculated using the CIEDE2000 formula between the photographic measurements and the manufacturer-provided reference values. A ΔE value of ≤ 2.4 was considered clinically acceptable, based on established perceptibility and acceptability thresholds reported in the literature.

## Materials and methods

Ethical considerations

Ethical approval for the study was obtained from the Institutional Ethics Committee of Chandra Dental College and Hospital, Barabanki, India (Ref No.: CDCH/PROSTH/2023-26/017) before commencement. Although this was an in vitro study, approval was obtained in accordance with institutional research guidelines.

Research design/study

This in vitro study was conducted in the Department of Prosthodontics, Chandra Dental College and Hospital, Barabanki, India, from June 1, 2025, to August 30, 2025. The study aimed to compare digital methods of dental shade selection using a smartphone (SP) camera and a digital single-lens reflex (DSLR) camera based on CIELAB color coordinates [1,2,5].

Sample selection

A commercial VITA 3D-Master shade guide comprising 26 shade tabs was used. All photographs were captured by a single trained operator to eliminate inter-operator variability. Images were obtained under natural daylight conditions on bright sunny days to maintain consistent illumination [2]. Shade guides from other manufacturers, images captured under cloudy conditions, and images obtained using flash illumination were excluded to minimize variability in color temperature and exposure [2,4].

Materials and equipment

The study utilized a smartphone camera (iPhone 13, Apple Inc., Cupertino, CA, USA), a DSLR camera (Canon EOS 700D, Canon Inc., Tokyo, Japan), a VITA 3D-Master shade guide (VITA Zahnfabrik, Bad Säckingen, Germany), a tripod stand for stabilization, a neutral gray card for color calibration, and a laptop computer (HP Inc., Palo Alto, CA, USA) for image processing.

Software

Digital image processing and color analysis were performed using Adobe Photoshop (Adobe Inc., San Jose, CA, USA), Color Lab.AI image analysis software, and CIEDE2000 color difference calculation software to obtain the CIELAB color coordinates (L*, a*, b*) and calculate color differences (ΔE) between photographic measurements and manufacturer reference values [6].

Study method/tools

The color temperature of illumination was maintained between 4000 and 5000 K to approximate standardized daylight conditions recommended for dental shade selection [4]. The illumination setup remained constant throughout the study period, and ambient light interference was minimized to ensure uniform lighting conditions. Both cameras were mounted on a tripod and positioned at a fixed distance of 18 cm from the shade tab to standardize image acquisition geometry. Angulation, framing, and background were maintained using a predefined protocol.

**Figure 1:**
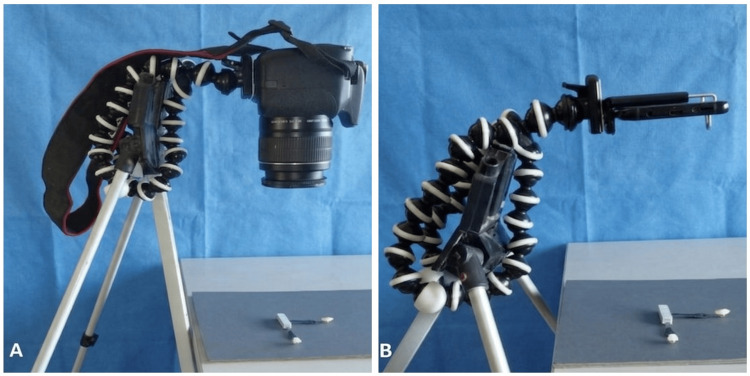
Standardized photographic setup for shade guide image acquisition. A: Digital single-lens reflex (DSLR) camera mounted on a tripod at a fixed distance and angulation for capturing images of the shade guide under controlled illumination conditions. B: Smartphone camera mounted on a tripod using a stabilizing adapter, positioned at the same standardized distance and angulation for image acquisition of the shade guide.

For each shade tab, two photographs were captured using each camera system, resulting in a total of 104 images. The most representative image was selected based on predefined objective criteria, including optimal focus, appropriate exposure, and absence of motion artifacts (Figure 1). Although this selection process may introduce a potential risk of bias, standardized criteria were applied to ensure consistency.

The DSLR camera settings were standardized (shutter speed: 1/125 s; aperture: f/22; ISO: 100; white balance: automatic; flash disabled), and images were captured in RAW format. Smartphone images were acquired in automatic mode with flash disabled to simulate routine clinical conditions, thereby enhancing external validity, although this may introduce variability compared with fully standardized DSLR settings. Smartphone images were captured in RAW (DNG) format alongside JPEG to preserve unprocessed image data and minimize the influence of in-camera processing algorithms. RAW images preserve original contrast, sharpness, and color information without compression, thereby enabling more reliable and reproducible color evaluation during subsequent analysis [7].

To minimize variability, all other parameters, including illumination, camera positioning, and environmental conditions, were kept constant throughout the study. To further enhance standardization, all images were captured by a single trained operator under identical environmental conditions and within a consistent time interval, minimizing operator-dependent and temporal variability.

Image analysis

Image selection was performed using predefined objective criteria, including optimal focus (edge sharpness), appropriate exposure (histogram without clipping), and absence of motion or imaging artifacts. However, the selection of a single image from two captures per shade tab may introduce a potential risk of selection bias.

Color analysis of DSLR images was conducted using Adobe Photoshop, following the protocol described by Bengel [7]. Each image was initially opened and evaluated using the information panel to obtain pixel-level color data. To eliminate global color cast, the levels adjustment tool was applied by selecting the neutral gray reference area using the midpoint eyedropper, thereby standardizing red (R), green (G), and blue (B) channel values. Subsequently, the image color space was converted from RGB to CIELAB, enabling quantitative assessment of L*, a*, and b* values using the histogram function.

The region of interest (ROI) corresponding to the central area of each shade tab was selected to ensure uniformity, while peripheral regions and specular reflections were excluded to avoid measurement bias. The CIELAB color coordinates (L*, a*, b*) for smartphone images were obtained using Color Lab.AI software, whereas DSLR-derived values were obtained using Adobe Photoshop, ensuring consistency within each imaging modality.

Color differences between photographic measurements and manufacturer-provided reference values were calculated using the CIEDE2000 color difference formula [6]. For interpretation, a threshold-based scoring system was applied: ΔE values < 2.4 were considered clinically imperceptible, whereas values ≥ 2.4 were considered perceptible to the human eye, in accordance with previously reported perceptibility thresholds in dental color science [8].

Image processing and color analysis

Digital image processing and color analysis were performed using Adobe Photoshop for DSLR images and Color Lab.AI for smartphone images. All images were analyzed in RAW format to minimize the effects of compression and in-camera processing. A neutral gray reference card (18% reflectance) was included in each image to standardize color calibration.

For DSLR images, Adobe Photoshop was used to obtain pixel-level RGB values, correct global color cast using the levels adjustment tool with a gray reference, and convert images into the CIELAB color space. Smartphone images were analyzed using Color Lab.AI, which enabled automated extraction of CIELAB (L*, a*, b*) coordinates. Identical calibration procedures were applied across both platforms to ensure consistency and comparability.

A standardized central ROI was selected for each shade tab, excluding peripheral areas and specular reflections. Mean CIELAB values were calculated from the selected region for each sample.

Color differences between photographic measurements and manufacturer reference values were calculated using the CIEDE2000 color difference formula (ΔE), which is widely accepted for assessing perceptibility and clinical acceptability in dental color science [6].

Outcome measures

The primary outcome measure was the mean color difference (ΔE) between photographic measurements and manufacturer reference values. A ΔE value of ≤ 2.4 was considered clinically acceptable.

Plan for data analysis

Data were recorded and analyzed using Microsoft Excel (Microsoft Corporation, Redmond, WA, USA) and SPSS version 23.0 (IBM Corporation, Armonk, NY, USA). Descriptive statistics, including mean, standard deviation, median, interquartile range, minimum, and maximum values of L*, a*, and b* parameters, were calculated for three groups: manufacturer values, DSLR values, and smartphone values.

The Shapiro-Wilk test was used to assess normality. As the data were not normally distributed, non-parametric tests were applied. The Friedman test was used for overall comparison among groups, followed by the Wilcoxon signed-rank test for pairwise comparisons. A p-value < 0.05 was considered statistically significant.

## Results

The CIELAB color coordinates of the VITA 3D-Master shade guide obtained from three sources, i.e., manufacturer reference values, DSLR camera measurements, and smartphone camera measurements, are presented in Table 1. The manufacturer's laboratory values served as the reference standard for comparison. Variations in the L*, a*, and b* coordinates were observed between the manufacturer values and those obtained from DSLR and smartphone images across the 26 shade tabs. Overall, the DSLR measurements showed closer agreement with the manufacturer's reference values, whereas the smartphone measurements demonstrated comparatively greater deviations.

**Table 1:** Comparison of Commission Internationale de l'Éclairage L*a*b* (CIELAB) color coordinates (L*, a*, b*) of the VITA 3D-Master shade guide obtained from the manufacturer's reference values, digital single-lens reflex (DSLR) camera, and smartphone (SP) camera measurements. CIELAB color coordinates (L*, a*, b*) of 26 shade tabs from the VITA 3D-Master shade guide. The manufacturer's laboratory values served as the reference standard, and the corresponding values obtained from images captured using a DSLR camera and a smartphone camera were compared to evaluate variations in color measurement across the different imaging methods.

VITA 3D-Master shade	Manufacturer lab values			DSLR camera values			Smartphone camera values		
	L* (unitless)	a* (unitless)	b* (unitless)	L* (unitless)	a* (unitless)	b* (unitless)	L* (unitless)	a* (unitless)	b* (unitless)
1M1	83.1	-0.1	12.5	83.5	0.4	13.3	82.4	0.5	14
1M2	84	-0.2	18.8	84.3	-0.5	20.1	83.5	0.3	16.8
2L1.5	79	0	18.5	78.5	1.1	18.9	79.9	1.9	17.4
2L2.5	79.5	0.2	24.5	78.2	0.8	23.8	82.2	1	22.7
2M1	78	0.8	14	78.5	2.3	13.5	78.6	2.9	14.5
2M2	78.7	0.9	19.9	79	1.2	21.7	80.7	1.9	17.3
2M3	79.2	0.7	25.3	79.3	1.1	25.1	79.7	2.4	25.2
2R1.5	77.8	1.5	16.3	77.3	2.6	16.4	78.2	2.8	16.1
2R2.5	79.5	1.7	23.3	79.7	2	24.9	79.2	2.7	22.2
3L1.5	73.1	1.5	20.3	74.4	2.1	19.9	74.7	2.9	19.1
3L2.5	73.9	1.9	26.2	73.8	2.9	26.2	74.1	3.1	26
3M1	73.4	1.8	15.4	73.6	2.9	15.9	73	3.5	14.7
3M2	74.6	2	21.5	74.8	2.9	20.9	75.1	3.5	22.2
3M3	75	2.6	27.9	75.6	3.5	28.1	76.3	4.1	27.7
3R1.5	73.4	2.7	17.9	73	3.3	18.3	74.4	4.1	17.1
3R2.5	73.6	3.5	25.9	73.5	4.1	26.1	72.9	4.9	26.5
4L1.5	69.2	2.8	21.7	68.3	3.3	21.6	68.1	4	20.9
4L2.5	69.1	3.7	28.5	69.5	3	28.4	68.6	5.5	28.3
4M1	68.3	2.9	17	68.6	3.4	17.4	68.7	4.5	16.6
4M2	70.1	3.7	23.7	70.5	2.9	23.8	71.1	4.8	24
4M3	69.5	4.8	30.7	70.1	3.9	30.4	68.6	6.7	29.8
4R1.5	69.6	4.3	20.8	69.1	4.2	21.7	68.7	4.9	19.6
4R2.5	69.2	5.1	26.3	68.8	5.4	26.9	68.2	6.1	27
5M1	64.4	4.2	19.4	64.7	3.7	18.9	64.8	5.1	20.1
5M2	65.1	5.7	26.3	66.5	5.3	26.4	67.7	6.3	26.7
5M3	65.9	7	33.4	66.5	6.1	33.1	67.7	7.6	33

Table 2 presents the CIELAB color coordinates of the VITA 3D-Master shade guide obtained from the manufacturer’s laboratory, DSLR camera, and smartphone camera measurements. For the L* value, the Friedman test revealed no statistically significant difference among the three measurement sources (χ² = 3.308, df = 2, P > 0.05), indicating comparable results for lightness across the methods. For the a* value, the Friedman test demonstrated a very highly significant difference among the groups (χ² = 40.923, df = 2, P < 0.001). Subsequent Wilcoxon signed-rank tests for pairwise comparisons revealed a significant difference between the manufacturer laboratory values and DSLR measurements (Z = -2.112, P < 0.05) and very highly significant differences between the manufacturer laboratory and smartphone measurements (Z = -4.461, P < 0.001) as well as between the DSLR and smartphone measurements (Z = -4.467, P < 0.001). For the b* value, the Friedman test showed no statistically significant difference among the measurement methods (χ² = 5.029, df = 2, P > 0.05).

**Table 2:** Comparison of L*, a*, and b* values of the VITA 3D-Master shade guide obtained from the manufacturer's laboratory, digital single-lens reflex (DSLR) camera, and smartphone camera measurements. L*, a*, and b* represent the color coordinates of the Commission Internationale de l'Éclairage L*a*b* (CIELAB) color system, where L* indicates lightness, a* represents the red–green axis, and b* represents the yellow–blue axis. The Friedman test was used to evaluate overall differences among the three measurement methods. When a statistically significant difference was detected, pairwise comparisons were performed using the Wilcoxon signed-rank test. A P-value < 0.05 was considered statistically significant. Values are expressed as χ² (chi-square), degrees of freedom (df), Z value, and corresponding P-value.

Statistical test	L* value (unitless)	a* value (unitless)	b* value (unitless)
Friedman test	χ² = 3.308, df = 2, P = 0.191 (>0.05), not significant	χ² = 40.923, df = 2, P < 0.001, very highly significant	χ² = 5.029, df = 2, P = 0.081 (>0.05), not significant
Wilcoxon signed-rank test (pairwise comparison)	Not applicable	Manufacturer laboratory vs. DSLR camera: Z = -2.112, P = 0.035 (<0.05), significant. Manufacturer laboratory vs. smartphone camera: Z = -4.461, P < 0.001, very highly significant. DSLR camera vs. smartphone camera: Z = -4.467, P < 0.001, very highly significant	Not applicable

The color difference (ΔE) values for the DSLR camera and smartphone camera measurements, using the manufacturer's laboratory values as the reference standard for the VITA 3D-Master shade guide, are presented in Table 3. Across the 26 shade tabs evaluated, the ΔE values obtained with the DSLR camera ranged from 0.39 to 2.00. In comparison, the ΔE values obtained with the smartphone camera ranged from 0.98 to 2.65. These findings indicate a relatively broader range of color difference for the smartphone camera measurements compared with the DSLR camera when referenced against the manufacturer's laboratory values.

**Table 3:** Color difference (ΔE) values for digital single-lens reflex (DSLR) camera and smartphone camera measurements using the manufacturer's laboratory values as the reference standard for the VITA 3D-Master shade guide. ΔE represents the color difference calculated between the values obtained from the manufacturer's laboratory (reference standard) and those measured using the DSLR camera and smartphone camera for each VITA 3D-Master shade tab. A lower ΔE value indicates a smaller color difference and closer agreement with the manufacturer's laboratory reference values. The table presents ΔE values for all 26 shade tabs of the VITA 3D-Master shade guide.

VITA 3D-Master shade	ΔE DSLR camera	ΔE smartphone camera
1M1	0.86	1.3
1M2	0.79	1.3
2L1.5	1.31	2.41
2L2.5	1.13	2.2
2M1	2	2.65
2M2	0.99	2.36
2M3	0.39	1.62
2R1.5	1.36	1.61
2R2.5	0.81	1.22
3L1.5	1.2	2.11
3L2.5	0.91	1.11
3M1	1.33	2.22
3M2	1.04	1.56
3M3	0.88	1.62
3R1.5	0.74	1.91
3R2.5	0.54	1.33
4L1.5	0.88	1.63
4L2.5	0.67	1.6
4M1	0.62	1.95
4M2	0.84	1.28
4M3	0.86	1.83
4R1.5	0.64	1.24
4R2.5	0.46	1.16
5M1	0.6	0.98
5M2	1.2	2.16
5M3	0.84	1.55

Table 4 presents the comparison of color difference (ΔE) values obtained from the DSLR camera and smartphone camera for the VITA 3D-Master shade guide. For the DSLR camera, the mean ± standard deviation (SD) of the ΔE values was 0.92 ± 0.34, with a median of 0.86 and an interquartile range (IQR) of 0.49. The observed values ranged from 0.39 to 2.00. For the smartphone camera, the mean ± SD of the ΔE values was 1.69 ± 0.45, with a median of 1.62 and an IQR of 0.83. The ΔE values ranged from 0.98 to 2.65. The Wilcoxon signed-rank test demonstrated a very highly significant difference between the ΔE values obtained from the DSLR camera and the smartphone camera (Z = -4.458, P < 0.001). The ΔE values were significantly higher for the smartphone camera compared with the DSLR camera.

**Table 4:** Comparison of color difference (ΔE) values between digital single-lens reflex (DSLR) camera and smartphone camera for the VITA 3D-Master shade guide. ΔE represents the color difference between the measurements obtained using the DSLR camera and smartphone camera when compared with the manufacturer's laboratory values (reference standard). Data are presented as mean ± standard deviation (SD), median with interquartile range (IQR), and minimum–maximum values. The Wilcoxon signed-rank test was used to compare ΔE values between the two camera systems. A P-value < 0.05 was considered statistically significant. Very highly significant indicates P < 0.001.

Statistical parameter	ΔE value	
	DSLR camera	Smartphone camera
Mean ± SD	0.92 ± 0.34	1.69 ± 0.45
Median (IQR)	0.86 (0.49)	1.62 (0.83)
Min-Max	0.39 - 2.00	0.98 - 2.65
Wilcoxon signed-rank test	Z = -4.458, P = 0.000 (<0.001), very highly significant	

Percentage differences in L*, a*, and b* values of the VITA 3D-Master shade guide obtained from the DSLR camera and smartphone camera measurements in comparison with the manufacturer's laboratory values are presented in Table 5. For the DSLR camera, the percentage difference ranged from -1.64% to 2.15% for L* values, -500.00% to 300.00% for a* values, and -3.57% to 9.05% for b* values. For the smartphone camera, the percentage difference ranged from -1.59% to 3.99% for L* values, -600.00% to 400.00% for a* values, and -13.07% to 12.00% for b* values. These findings indicate a comparatively wider variation in percentage differences for the smartphone camera measurements than for the DSLR camera when referenced against the manufacturer's laboratory values.

**Table 5:** Percentage difference of L*, a*, and b* values from the manufacturer's laboratory values for the VITA 3D-Master shade guide obtained using digital single-lens reflex (DSLR) camera and smartphone camera measurements. The table presents the percentage differences in Commission Internationale de l'Éclairage L*a*b* (CIELAB) color parameters (L*, a*, and b*) for each of the 26 shade tabs of the VITA 3D-Master shade guide obtained from DSLR camera and smartphone camera measurements, using the manufacturer's laboratory values as the reference standard.

VITA 3D-Master shade	DSLR camera values			Smartphone camera values		
	L* (unitless)	a* (unitless)	b* (unitless)	L* (unitless)	a* (unitless)	b* (unitless)
1M1	0.48	-500.00	6.40	-0.84	-600.00	12.00
1M2	0.36	150.00	6.91	-0.60	-250.00	-10.64
2L1.5	-0.63	0.00	2.16	1.14	0.00	-5.95
2L2.5	-1.64	300.00	-2.86	3.40	400.00	-7.35
2M1	0.64	187.50	-3.57	0.77	262.50	3.57
2M2	0.38	33.33	9.05	2.54	111.11	-13.07
2M3	0.13	57.14	-0.79	0.63	242.86	-0.40
2R1.5	-0.64	73.33	0.61	0.51	86.67	-1.23
2R2.5	0.25	17.65	6.87	-0.38	58.82	-4.72
3L1.5	1.78	40.00	-1.97	2.19	93.33	-5.91
3L2.5	-0.14	52.63	0.00	0.27	63.16	-0.76
3M1	0.27	61.11	3.25	-0.54	94.44	-4.55
3M2	0.27	45.00	-2.79	0.67	75.00	3.26
3M3	0.80	34.62	0.72	1.73	57.69	-0.72
3R1.5	-0.54	22.22	2.23	1.36	51.85	-4.47
3R2.5	-0.14	17.14	0.77	-0.95	40.00	2.32
4L1.5	-1.30	17.86	-0.46	-1.59	42.86	-3.69
4L2.5	0.58	-18.92	-0.35	-0.72	48.65	-0.70
4M1	0.44	17.24	2.35	0.59	55.17	-2.35
4M2	0.57	-21.62	0.42	1.43	29.73	1.27
4M3	0.86	-18.75	-0.98	-1.29	39.58	-2.93
4R1.5	-0.72	-2.33	4.33	-1.29	13.95	-5.77
4R2.5	-0.58	5.88	2.28	-1.45	19.61	2.66
5M1	0.47	-11.90	-2.58	0.62	21.43	3.61
5M2	2.15	-7.02	0.38	3.99	10.53	1.52
5M3	0.91	-12.86	-0.90	2.73	8.57	-1.20

Comparison of the percentage difference in L* values for the DSLR camera and smartphone camera measurements of the VITA 3D-Master shade guide is presented in Table 6. For the DSLR camera, the mean ± SD of the L* percentage difference was 0.19 ± 0.84, with a median of 0.31 and an interquartile range (IQR) of 1.15. The observed values ranged from -1.64 to 2.15. For the smartphone camera, the mean ± SD was 0.57 ± 1.54, with a median of 0.60 and an IQR of 2.26. The percentage difference ranged from -1.59 to 3.99. The Wilcoxon signed-rank test indicated that the difference in the percentage difference of L* values between the DSLR camera and smartphone camera was not statistically significant (Z = -0.978, P > 0.05).

**Table 6:** Comparison of percentage difference in L* values for digital single-lens reflex (DSLR) camera and smartphone camera measurements of the VITA 3D-Master shade guide. The table presents the comparison of percentage differences in L* (lightness) values for DSLR camera and smartphone camera measurements of the VITA 3D-Master shade guide, using the manufacturer's laboratory values as the reference standard. Data are expressed as mean ± standard deviation (SD), median with interquartile range (IQR), and minimum–maximum values. The Wilcoxon signed-rank test was applied to compare the percentage differences between the two camera systems. A P-value < 0.05 was considered statistically significant.

Statistical parameter	DSLR camera L* (unitless)	Smartphone camera L* (unitless)	Wilcoxon signed-rank test
Mean ± SD	0.19 ± 0.84	0.57 ± 1.54	-
Median (IQR)	0.31 (1.15)	0.60 (2.26)	-
Min–Max	-1.64 - 2.15	-1.59 - 3.99	-
Z value	-	-	-0.978
P-value	-	-	0.328 (>0.05)
Significance	-	-	Not significant

Comparison of the percentage difference in a* values for the DSLR camera and smartphone camera measurements of the VITA 3D-Master shade guide is presented in Table 7. For the DSLR camera, the mean ± SD of the a* percentage difference was 20.74 ± 128.19, with a median of 17.75 and an IQR of 62.00. The observed values ranged from -500.00 to 300.00. For the smartphone camera, the mean ± SD was 41.44 ± 171.44, with a median of 50.25 and an IQR of 70.14. The percentage difference ranged from -600.00 to 400.00. The Wilcoxon signed-rank test revealed that the difference in the percentage difference of a* values between the DSLR camera and smartphone camera was highly significant (Z = -3.095, P < 0.01). The percentage difference in the a* values was significantly higher in the smartphone camera compared with the DSLR camera.

**Table 7:** Comparison of percentage difference in a* values for digital single-lens reflex (DSLR) camera and smartphone camera measurements of the VITA 3D-Master shade guide. The table presents the comparison of percentage differences in a* values (red–green chromatic axis) obtained from DSLR camera and smartphone camera measurements of the VITA 3D-Master shade guide, using the manufacturer's laboratory values as the reference standard. Data are expressed as mean ± standard deviation (SD), median with interquartile range (IQR), and minimum–maximum values. The Wilcoxon signed-rank test was applied to evaluate differences between the two camera systems. A P-value < 0.05 was considered statistically significant.

Statistical parameter	DSLR camera a* (unitless)	Smartphone camera a* (unitless)	Wilcoxon signed-rank test
Mean ± SD	20.74 ± 128.19	41.44 ± 171.44	-
Median (IQR)	17.75 (62.00)	50.25 (70.14)	-
Min–Max	-500.00 - 300.00	-600.00 - 400.00	-
Z value	-	-	-3.095
P-value	-	-	0.002 (<0.01)
Significance	-	-	Highly significant

Comparison of the percentage difference in b* values for the DSLR camera and smartphone camera measurements of the VITA 3D-Master shade guide is presented in Table 8. For the DSLR camera, the mean ± SD of the b* percentage difference was 1.21 ± 3.30, with a median of 0.52 and an IQR of 3.49. The observed values ranged from -3.57 to 9.05. For the smartphone camera, the mean ± SD was -1.78 ± 5.10, with a median of -1.21 and an IQR of 6.70. The percentage difference ranged from -13.07 to 12.00. The Wilcoxon signed-rank test indicated that the difference in the percentage difference of b* values between the DSLR camera and smartphone camera was not statistically significant (Z = -1.918, P > 0.05).

**Table 8:** Comparison of percentage difference in b* values for digital single-lens reflex (DSLR) camera and smartphone camera measurements of the VITA 3D-Master shade guide. The table presents the comparison of percentage differences in b* values (yellow–blue chromatic axis) obtained from DSLR camera and smartphone camera measurements of the VITA 3D-Master shade guide, using the manufacturer's laboratory values as the reference standard. Data are expressed as mean ± standard deviation (SD), median with interquartile range (IQR), and minimum–maximum values. The Wilcoxon signed-rank test was applied to evaluate differences between the two camera systems. A P-value < 0.05 was considered statistically significant.

Statistical parameter	DSLR camera b* (unitless)	Smartphone camera b* (unitless)	Wilcoxon signed-rank test
Mean ± SD	1.21 ± 3.30	-1.78 ± 5.10	-
Median (IQR)	0.52 (3.49)	-1.21 (6.70)	-
Min–Max	-3.57 - 9.05	-13.07 - 12.00	-
Z value	-	-	-1.918
P-value	-	-	0.055 (>0.05)
Significance	-	-	Not significant

DSLR measurements demonstrated superior agreement with manufacturer reference values compared with smartphone measurements.

## Discussion

The restoration of dental form, function, and esthetics represents a fundamental objective of restorative dentistry, with accurate shade selection being a critical determinant of esthetic success. Even minor discrepancies in tooth color can compromise the visual integration of restorations, particularly in the anterior region. However, shade selection remains challenging due to the subjective nature of human color perception and the influence of multiple variables, including lighting conditions, operator experience, and environmental factors [1,2,9].

Conventional visual shade matching using shade guides is widely practiced but is associated with considerable variability. This variability arises from differences in individual color perception and external conditions affecting visual assessment [1,10]. Consequently, digital technologies such as spectrophotometers, colorimeters, and digital photography have been introduced to improve objectivity and reproducibility in shade determination [2,11].

Digital photography has emerged as a valuable adjunct for shade communication, enabling the capture of detailed images alongside reference shade tabs. When standardized protocols are followed, including the use of gray reference cards, controlled illumination, and RAW image capture, digital imaging can provide reproducible color information suitable for quantitative analysis [11,12].

Recent advancements in smartphone camera technology have introduced a convenient alternative to DSLR cameras. Smartphones offer advantages such as portability, ease of use, and rapid image transfer, facilitating efficient communication with dental laboratories [13]. However, concerns remain regarding their accuracy and reliability for precise color determination.

In the present study, color measurements obtained from DSLR and smartphone images were compared with the manufacturer's reference values using CIELAB coordinates. The results demonstrated that DSLR images exhibited lower color differences ΔE compared with smartphone images, indicating closer agreement with reference values. The mean ΔE value for DSLR images was within the clinically acceptable threshold (≤2.4), whereas some smartphone measurements exceeded this limit, suggesting reduced color accuracy [14].

Statistical analysis revealed a significant difference between the two imaging systems (P < 0.001), indicating that DSLR cameras performed superiorly under standardized conditions. Analysis of individual CIELAB parameters showed that significant differences were primarily associated with the a* parameter, whereas L* and b* values did not differ significantly. The greater variability observed in the a* parameter may be attributed to differences in sensor sensitivity and internal image processing algorithms, particularly in smartphone cameras, which often apply automatic color enhancement.

These findings are consistent with previous studies reporting variability in color representation across different imaging devices and supporting the reliability of DSLR photography when standardized protocols are employed [12,15].

The greater percentage variation observed in the a* parameter compared with L* and b* values may also be influenced by the relatively small baseline values of a*, which can exaggerate percentage differences. Additionally, lighter shades within the VITA 3D-Master system demonstrated increased chromatic variability, likely due to smaller absolute color differences in these shades.

The results of this study support the role of digital photography as a useful tool in restorative dentistry for shade documentation and communication. While smartphone cameras offer practical advantages, DSLR systems demonstrated superior color accuracy under controlled conditions.

However, the clinical superiority of DSLR photography over smartphone imaging cannot be definitively established without further in vivo investigations.

Several limitations should be considered when interpreting these findings. Despite efforts to standardize illumination, minor variations in daylight conditions may have influenced image capture. Additionally, the study was conducted under in vitro conditions using shade tabs rather than intraoral environments, where factors such as saliva, soft tissues, and patient movement may affect color perception.

Smartphone imaging was performed using automatic settings to simulate clinical practice; however, this may introduce variability due to device-dependent processing algorithms. Furthermore, the selection of a single image from two captures per shade tab may introduce potential selection bias. The study also utilized a single operator, and interoperator variability was not assessed.

Future research should focus on evaluating smartphone-based shade selection under clinical conditions and on developing standardized imaging protocols to enhance the reliability of mobile imaging systems in dentistry.

The present study was conducted under controlled in vitro conditions, which, although advantageous for standardization, do not fully replicate the complexity of the intraoral environment. Factors such as saliva, soft tissue influence, adjacent tooth structures, and varying illumination conditions may affect shade perception and selection in clinical practice. Therefore, caution must be exercised when extrapolating these findings directly to in vivo situations. Nevertheless, the in vitro design allowed for precise control of variables and reliable comparison between materials, thereby providing valuable baseline data. Similar methodological approaches have been employed in previous studies to ensure reproducibility; however, clinical studies have reported variability in shade matching due to intraoral factors. Future research should focus on in vivo evaluation and the incorporation of digital shade-matching technologies to validate these findings under clinical conditions.

## Conclusions

Within the limitations of this in vitro study, DSLR camera-based imaging demonstrated closer agreement with manufacturer reference values compared with smartphone-based imaging for dental shade assessment. Using a clinically acceptable color difference threshold of ΔE ≤ 2.4, a greater proportion of DSLR measurements fell within acceptable limits (approximately 75%) compared with smartphone measurements (approximately 55%). Under standardized conditions, DSLR imaging exhibited superior accuracy and consistency in color reproduction. However, these findings should be interpreted with caution, as the study was conducted under controlled extraoral conditions using shade tabs, and smartphone imaging was performed in automatic mode, which may introduce variability. Therefore, while DSLR imaging appears more reliable for shade determination, the clinical applicability of these findings remains limited. Further in vivo studies are required to validate the performance of both DSLR and smartphone imaging systems under real clinical conditions.
